# Serum albumin-fatty acid saturation test

**DOI:** 10.1016/j.mex.2019.08.004

**Published:** 2019-08-24

**Authors:** Cassiano Felippe Gonçalves-de-Albuquerque, Marcos Roberto Colombo Barnese, Mariana Alves Soares, Mauro Velho Castro Faria, Adriana Ribeiro Silva, Hugo Caire Castro Faria Neto, Patrícia Burth, Mauricio Younes-Ibrahim

**Affiliations:** a Laboratório Integrado de Nefrologia, Department of Internal Medicine, Medical Sciences School, State University of Rio de Janeiro, Brazil; b Laboratório de Imunofarmacologia, Oswaldo Cruz Institute, Fiocruz, Rio de Janeiro, Brazil; c Laboratório de Enzimologia e Sinalização Celular, Department of Cellular and Molecular Biology, Federal Fluminense University, Niteroi, Brazil; d Laboratório de Imunofarmacologia, Departamento de Bioquímica, Universidade Federal do Estado do Rio de Janeiro, Rio de Janeiro, RJ 20211-010, Brazil; e Retired, Previously at Laboratório Integrado de Nefrologia, Department of Internal Medicine, Medical Sciences School, State University of Rio de Janeiro, Brazil

**Keywords:** Serum albumin-fatty acid saturation test, Albumin, Non-esterified fatty acids, Lipotoxicity

## Abstract

Circulating non-esterified fatty acids (NEFA) are toxic to mammalian cells. They increase in diseases such as diabetes and sepsis. Herein we propose a serum albumin-fatty acid saturation test.

• We based our test on three methodologies: isoelectric focusing (IF) of human plasma albumin, staining proteins after isoelectric focusing in gels with Coomassie Brilliant Blue, and serum albumin measurement with bromocresol green.
• The test consists in the determination of albumin IF and staining with bromocresol green. If albumin is saturated with NEFA, it focuses on lower pH, meaning it is the threshold to bind to them. Excessive NEFA is free and toxic. Many other tests are available for NEFA quantification as NEFA kit assay. All colorimetric assays are used for quantification of NEFA and other tests need expensive equipment to read out the results, and they do not measure albumin levels.
• Our method focused on albumin-NEFA saturation instead of just NEFA quantification. Critically ill patients have an alteration in both albumin and NEFA. Therefore, our test undergoes less daytime variation compared to assays that measure absolute NEFA values, allowing a more reliable use as an indicator of albumin-fatty acid saturation and NEFA toxicity.

## Graphical abstract

**Figure:**
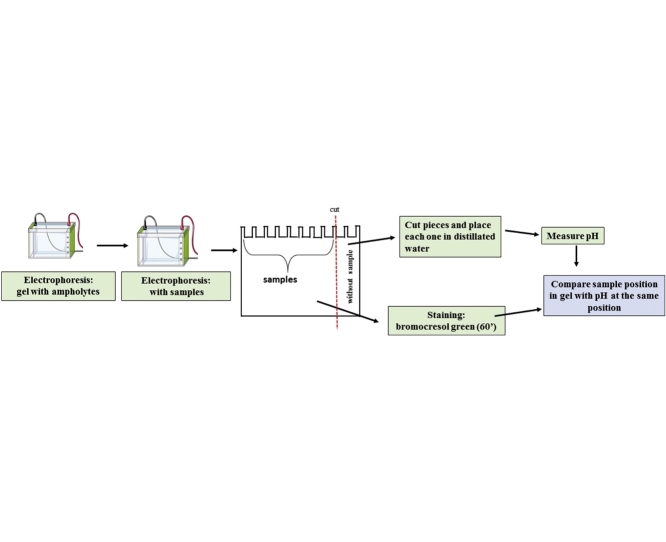

Specifications Table

**Table:** 

Subject Area:	Medicine and Dentistry
More specific subject area:	Biochemistry
Method name:	Serum albumin-fatty acid saturation test
Name and reference of original method:	S.P. Basu, S.N. Rao, J.A. Hartsuck, Influence of fatty acid and time of focusing on the isoelectric focusing of human plasma albumin, Biochimica et biophysica acta 533(1) (1978) 66-73.
	B.T. Doumas, W.A. Watson, H.G. Biggs, Albumin standards and the measurement of serum albumin with bromcresol green, Clin Chim Acta 31(1) (1971) 87-96.
	O. Vesterberg, L. Hansen, A. Sjosten, Staining of proteins after isoelectric focusing in gels by new procedures, Biochimica et biophysica acta 491(1) (1977) 160-6.
Resource availability:	N/A

## Method details

**Fig. 1:**
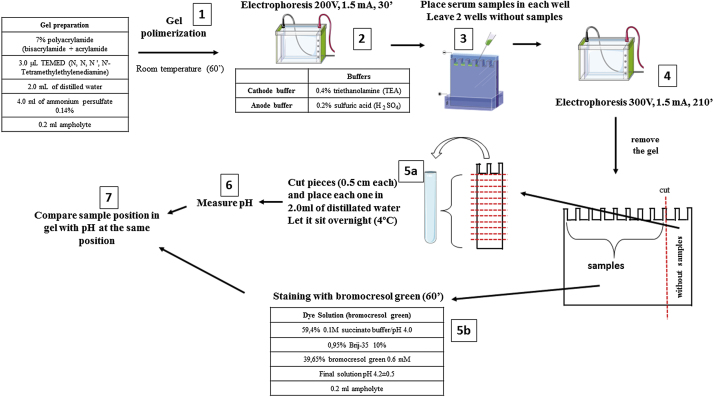
Experimental schematic drawing. The first step is gel polymerization with the first electrophoresis (1 and 2). Next step is sample application and second electrophoresis (3 and 4). Cut the gel in two pieces, one with two lanes without samples and cut it in pieces and place them in distilled water (5a) and stain the samples in the second gel piece (5b). After measuring pH (6) compare the stained sample’s position with pH at the same position (7).

**Fig. 2:**
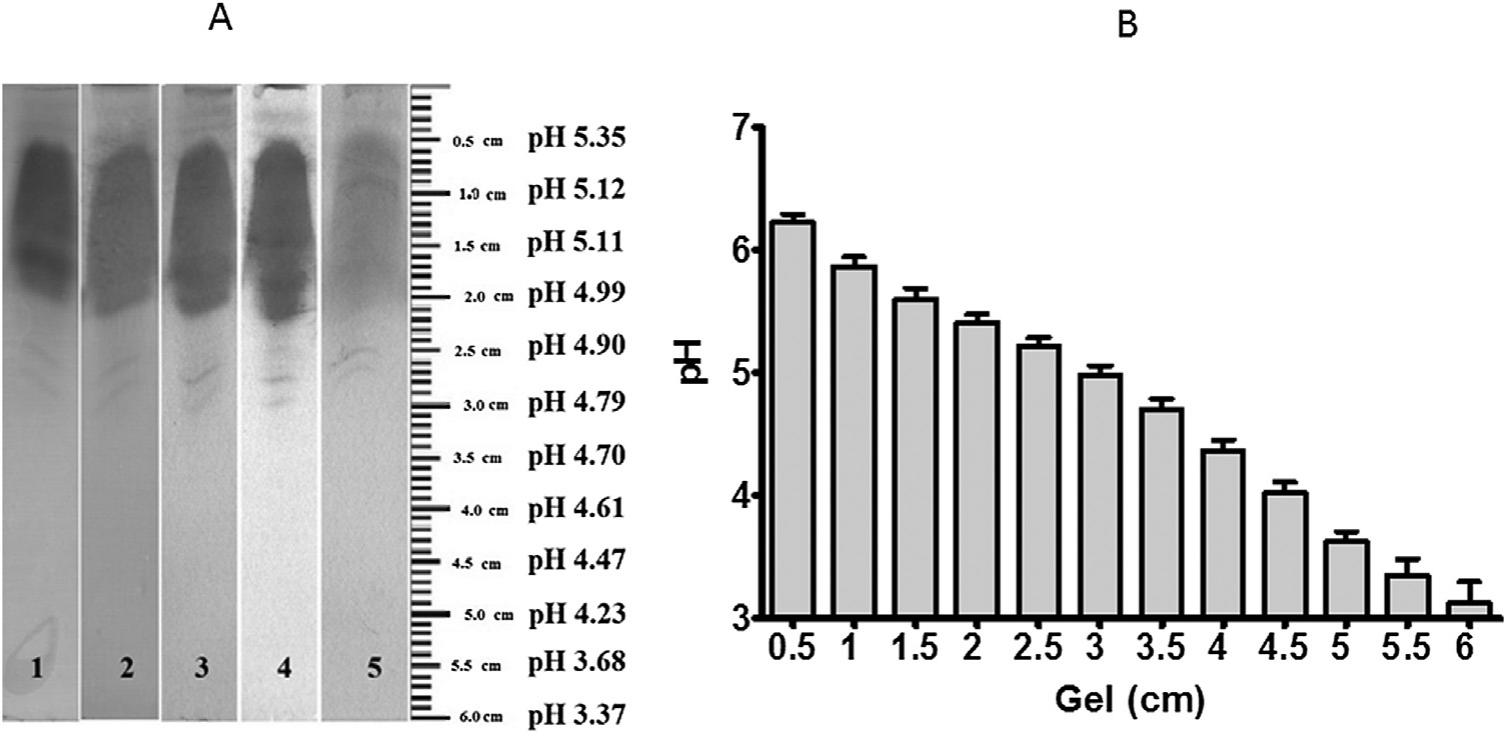
Isoelectric focalization images. Isoelectric focalization of albumin from five controls (A). The samples were processed according to MM. Albumin from all samples focalized at pH 5.11. Assessment of pH in each 0.5 cm gel piece. The bars represent the pH gradient at the end of isoelectric focusing of 7 independent runs. The data is represented as median ± standard error.

Herein we adapted 3 methodologies, isoelectric focusing of human plasma albumin [1], staining proteins after isoelectric focusing in gels with Coomassie Brilliant Blue R 250 [2], and measurement of serum albumin with bromocresol green [3], in one. NEFA assays kits require expensive equipment such as microplate reader, and other available methods require high-performance liquid chromatography and/or mass spectrometry. Our test only uses electrophoresis equipment. We used samples from critically ill patients with non-esterified fatty acid alteration and healthy volunteers. We showed a difference in the pattern of albumin isoelectric focusing in ill patients compared to the control group [4]. It consists of staining human serum albumin with bromocresol green after isoelectric focusing in order to detect albumin-fatty acid saturation. We chose the isoelectric focusing method (IF) because albumin molecules, depending on the number of fatty acids bound, can acquire a new effective electric charge (final load of fatty acids with protein) and also new conformation [2]. Then, depending on its saturation with fatty acids, the albumin acquires new isoelectric points that can be analyzed by the IF. Excessive NEFA is free and toxic – only 7 fatty acid molecules can bind to albumin at once. For IF we based on Basu et al. [1], briefly gel preparation used 2.0 ml of 7% polyacrylamide (bisacrylamide + acrylamide); 3.0 μL TEMED (*N*, *N*, *N* ', *N*'-Tetramethylethylenediamine); 2.0 ml of distilled water; 4.0 ml of ammonium persulfate 0.14%; 0.2 ml ampholyte (Ampholyte High Resolution pH 4–6) (all reagents were purchased from Sigma-Aldrich) (Fig. 1 step 1). Two buffers were prepared, the cathode buffer was 0.4% triethanolamine (TEA) and the anode buffer was 0.2% sulfuric acid (H _2_ SO_4_) (Sigma-Aldrich). After polymerization, the gel was placed in the electrophoretic cell without the samples, and subjected to 200 V, amperage 1.5 mA for 30 min, to form the pH gradient on the gel (Fig. 1 step 2). For the electrophoretic run, a power source of the brand Incibras was used, that develops voltage up to 1000 V. After 30 min of this initial run, samples from control sera or from patients were placed in their respective wells (Fig. 1 step 3). Some wells were left without samples to measure the gel pH, and hence, trace the pH gradient. We perform a second run with samples (210 min at a voltage 300 V and amperage of 1.5 mA) (Fig. 1 step 4). After that, the gel with the samples was stained with a high-affinity albumin dye, bromocresol green (according to modification of the albumin dosage method of Doumas et al. [3]) (Fig. 1 step 5b). This dying method is usually employed for the determination of serum albumin or plasma, but here we used to staining the albumin in the gel. This dye primarily targets albumin, although some serum alpha-proteins may be interfering. Such dye adapted for the staining of albumin in the gels contained 59.4% 0.1 M succinate buffer/pH 4.0; 0.95% Brij-35 (detergent) 10%; 39.65% bromocresol green 0.6 mM (Sigma-Aldrich). The final working solution should have a pH of 4.2 ± 0.5. The gel remained in contact with the dye for 1 h. The dying process needs care to properly function. The image capture must be done 1 h after the fresh dye addition because the light exposure faints the band. In these optimal conditions, the stain works very nicely. For example, we showed 5 samples from healthy volunteers (Fig. 2A). The second part of the gel without samples was cut in small pieces (12 pieces of 0.5 cm in length each) (Fig. 1 step 5a). We placed each piece in a glass tube with 2.0 ml of distilled water. The tubes were stored at 4 °C overnight and the pH was measured the next day in pH meter, to define the pH gradient formed (Fig. 1 step 6 and Fig. 2b).

## Method validation

Albumin isoelectric focusing in serum samples from critically ill patients and controls was compared with NEFA/albumin ratios measured by HPLC [4]. This test may indicate to physicians the potential NEFA lipotoxicity [5], guiding them throughout better patient management. The albumin saturation test can point out serum albumin-NEFA saturation through a cheap assay that could be performed by any care facility. In isoelectric focusing the molecules are separated according to their effective electric charge [3] and NEFA bound to human serum albumin change isoelectric focusing [1]. Depending on the amount of fatty acid (FA) bound to the protein, it acquires a new effective electric charge and molecular conformation. Isoelectric focusing plasma albumin in preeclamptic women in lower pH is due to NEFA binding in these subjects [6]. In our experiments, we also observed a shift to acidic pH in IF of serum albumin in critically ill patients likely due to NEFA-saturated albumin. NEFA levels can vary during day time [7]. With this method, we can assay not only NEFA levels measured by colorimetric assays but the albumin-NEFA saturation. The ration between albumin/NEFA is critical because patients with lower levels of serum albumin suffer greatly with elevations on serum NEFA. In our opinion, it is due to the lower capacity of albumin to bind NEFA avoiding its deleterious effects such as mitochondrial dysfunction and lipoapoptosis [[8], [9], [10]]. In previous work, we showed the crucial role of ration NEFA/albumin in determine NEFA toxic where higher ration higher risk of NEFA toxicity [4]. The albumin-NEFA saturation test proposed here provides information concerning albumin-NEFA saturation determined by albumin isoelectric focusing and staining with bromocresol green. Thus this assay could be a handy alternative to current methodology for serum NEFA quantification with the advantage of measure albumin ability to bind NEFA.
